# Admixture mapping in the Hispanic Community Health Study/Study of Latinos reveals regions of genetic associations with blood pressure traits

**DOI:** 10.1371/journal.pone.0188400

**Published:** 2017-11-20

**Authors:** Tamar Sofer, Leslie J. Baier, Sharon R. Browning, Timothy A. Thornton, Gregory A. Talavera, Sylvia Wassertheil-Smoller, Martha L. Daviglus, Robert Hanson, Sayuko Kobes, Richard S. Cooper, Jianwen Cai, Daniel Levy, Alex P. Reiner, Nora Franceschini

**Affiliations:** 1 Division of Sleep and Circadian Disorders, Brigham and Women’s Hospital, Boston, MA, United States of America; 2 Department of Medicine, Harvard Medical School, Boston, MA, United States of America; 3 Phoenix Epidemiology and Clinical Research Branch, NIDDK, NIH, Phoenix, AZ, United States of America; 4 Department of Biostatistics, University of Washington, Seattle, WA, United States of America; 5 Division of Health Promotion and Behavioral Science, San Diego State University, San Diego, CA, United States of America; 6 Department of Epidemiology and Population Health, Albert Einstein College of Medicine, Bronx, NY, United States of America; 7 Feinberg School of Medicine, Northwestern University, Chicago, IL, United States of America; 8 Institute for Minority Health Research, University of Illinois at Chicago, Chicago, IL, United States of America; 9 Department of Public Health Sciences, Stritch School of Medicine, Loyola University Chicago, Chicago, IL, United States of America; 10 Department of Biostatistics, University of North Carolina, Chapel Hill, NC, United States of America; 11 The Framingham Heart Study, Framingham, MA, United States of America; 12 Population Sciences Branch, National Heart, Lung, and Blood Institute, National Institutes of Health, Bethesda, MD, United States of America; 13 Division of Public Health Sciences, Fred Hutchinson Cancer Research Center, Seattle, WA, United States of America; 14 Department of Epidemiology, University of North Carolina, Chapel Hill, NC, United States of America; University of Texas Health Science Center at San Antonio, UNITED STATES

## Abstract

Admixture mapping can be used to detect genetic association regions in admixed populations, such as Hispanics/Latinos, by estimating associations between local ancestry allele counts and the trait of interest. We performed admixture mapping of the blood pressure traits systolic and diastolic blood pressure (SBP, DBP), mean arterial pressure (MAP), and pulse pressure (PP), in a dataset of 12,116 participants from the Hispanic Community Health Study/Study of Latinos (HCHS/SOL). Hispanics/Latinos have three predominant ancestral populations (European, African, and Amerindian), for each of which we separately tested local ancestry intervals across the genome. We identified four regions that were significantly associated with a blood pressure trait at the genome-wide admixture mapping level. A 6p21.31 Amerindian ancestry association region has multiple known associations, but none explained the admixture mapping signal. We identified variants that completely explained this signal. One of these variants had *p*-values of 0.02 (MAP) and 0.04 (SBP) in replication testing in Pima Indians. A 11q13.4 Amerindian ancestry association region spans a variant that was previously reported (*p*-value = 0.001) in a targeted association study of Blood Pressure (BP) traits and variants in the vitamin D pathway. There was no replication evidence supporting an association in the identified 17q25.3 Amerindian ancestry association region. For a region on 6p12.3, associated with African ancestry, we did not identify any candidate variants driving the association. It may be driven by rare variants. Whole genome sequence data may be necessary to fine map these association signals, which may contribute to disparities in BP traits between diverse populations.

## Introduction

Cardiovascular disease is a major cause of death in the United States and worldwide. Blood pressure is a strong risk factor for cardiovascular disease and has a known genetic component. Multiple genome-wide association studies (GWASs) have been carried out in populations of European [1–4], Asians/East Asian, [5] and African [6–8] ancestries, in Hispanics/Latinos, [9, 10] including the Hispanic Community Health Study/Study of Latinos (HCHS/SOL), and also in trans-ethnic settings. [11–14] GWAS tests the association of each “dose” of genotyped or imputed variant with a trait of interest. In populations such as Hispanics/Latinos who are an admixture of European, African, and Amerindian ancestries, association studies can be performed using an admixture mapping approach, where one tests the association of local ancestry counts in genomic intervals spanning tens, or even hundreds of thousands of base pairs, and the trait. Admixture mapping is especially useful for analysis of blood pressure traits, because their distribution differ between ancestral groups. [15]

In the simplest setting, the admixture mapping effect is equal to the effect of a causal genotype within the local ancestry interval (LAI), down-weighted by the difference in the causal genotype’s allele frequencies between ancestries. The admixture mapping effect also relates to the difference in the causal variant’s effect size between ancestries, if exists (see S1 File for details). Admixture mapping can be more powerful than association testing [16] in the sense that a statistically significant association may be detected in admixture mapping but not in association mapping, in some settings: (1) when a genotype is not typed or imputed in the genotyping platform, but is found within and LAI—this is especially true for rare variants that are typed less (or are filtered out due to unreliable statistical properties; (2) there are multiple associated variants within an LAI; and (3) the number of LAIs is smaller than the number of genotypes tested in a GWAS, leading to reduced multiple testing burden. However, a follow-up step of searching for causal variants within associated LAIs is also needed.

We performed admixture mapping of quantitative blood pressure traits, SBP, DBP, MAP, and PP in a population of United States (U.S.) Hispanics/Latinos from the HCHS/SOL. We leveraged the background groups of the HCHS/SOL (Central American, Cuban, Dominican, Mexican, Puerto Rican, South American) to fine-map candidate association variants from detected LAIs, which we then followed-up with conditional analysis, to verify that they indeed account for the admixture mapping associations. We assessed replication of four Amerindian variant associations in a population of Southwest American Indians.

## Materials and methods

### The Hispanic Community Health Study/Study of Latinos

The HCHS/SOL [17, 18] is a longitudinal cohort study of US Hispanics/Latinos, with first visits taking place during 2008–2011. Participants were recruited in four field centers (Bronx, NY, Chicago, IL, Miami, FL, and San Diego, CA) by a two-stage sampling design in which census block units were sampled at a first stage, followed by household sampling at a second stage. Either single or multiple participants were sampled from each household. The sampling was preferential towards areas with high proportions of Hispanics/Latinos. The IRB committees for the HCHS Coordinating Center at UNC Chapel Hill, San Diego State University, University of Illinois at Chicago, University of Miami, and Yeshiva University-Albert Einstein College of Medicine have all reviewed and approved the informed consent documents and study protocol. Written and signed informed consents in the language preferred by the participants are administered and archived at each of the participating field centers. All participants in this publication from HCHS/SOL have consented to use of their genetic and non-genetic data. Anyone not providing consent has been excluded from this analysis. Of the study individuals, 12,116 consented for participation in genetic studies and were eligible to participate in the present study after application of quality control and exclusion criteria.

### Blood pressure outcomes

We defined and used blood pressure outcomes as in the blood pressure GWAS study [10], with the additional exclusions of 165 individuals that withdrew consent for participation in genetic studies. Detailed description of the blood pressure measurement procedure is provided elsewhere. [19] In brief, we analyzed four quantitative blood pressure outcomes based on participants’ first clinic visit: visit: SBP and DBP, PP (SBP-DBP), and MAP (DBP-1/3PP). For individuals taking anti-hypertensive medications, we added 5 mmHg to measured DBP values and 10 mmHg to SBP measured values.

### Exclusion criteria

For all outcomes, we excluded 95 individuals with inconsistencies in their measured SBP or DBP (Omron mean and mean of raw measures difference ≥ 5mmHg), 19 individuals with high degree of Asian ancestry (detected and reported in [20]), 13 individuals without an associated background group (see below), 328 individuals with missing covariates or outcomes, and 70 individuals with either SBP<80 or DBP < 50. We removed a single individual with negative PP value and winsorized two outlying extreme values to have the value of the mean +6 standard deviations of PP, calculated on the analyzed sample set. Compared to the GWAS reported in [10], this study sample had 162 fewer individuals, because these HCHS/SOL participants withdrew their consent for genetic studies.

### Genotyping

Genotyping and quality control for HCHS/SOL have been described in Conomos et al. (2016) [20]. In brief, consenting HCHS/SOL participants were genotyped on the HCHS/SOL custom Illumina 15041502 B3 array. The custom array comprised the Illumina Omni 2.5M array (HumanOmni2.5-8v.1-1) ancestry-informative markers; known GWAS SNPs and drug absorption, distribution, metabolism, and excretion (ADME) markers, and additional custom content including ∼150,000 SNPs selected from the CLM (Colombian in Medellin, Colombia), MXL (Mexican Ancestry in Los Angeles, California), and PUR (Puerto Rican in Puerto Rico) samples in the 1000 Genomes phase 1 data to capture a greater amount of Amerindian genetic variation. [21]

We applied standardized quality-assurance and quality-control methods [22] to generate recommended SNP- and sample-level quality filters. Samples were checked for sex discrepancies, gross chromosomal anomalies, relatedness and population structure, missing call rates, batch effects, and duplicate-sample discordance. SNPs were checked for Hardy-Weinberg equilibrium, minor allele frequency (MAF), duplicate-probe discordance, Mendelian errors, and missing call rate. A total of 12,803 unique study participants passed QC and met specific clinical inclusion criteria. A total of 2,232,944 SNPs passed filters for both quality and informativeness (polymorphic and unduplicated) and were carried forward for imputation, analysis-specific exclusion criteria described above including the exclusion of 162 who withdrew their consent for genetic studies, and finally, downstream association analyses.

### Genetic structure inference: Genetic analysis groups and local ancestry

HCHS/SOL participants self-identified as primarily associated with one of six background groups: Central American, Cuban, Dominican, Mexican, Puerto Rican, and South American. Based on these groups, “genetic analysis groups” were constructed as described in Conomos et al. (2016) [20]. The genetic analysis groups mostly overlap self-identified background groups, but were constructed to be more genetically homogeneous, as determined using PC analysis, and to assign groups for individuals who self-identified as having “more than one” or “other” background. Of these groups, the Mainland groups (Mexicans, Central Americans, and South Americans) have high proportion of Amerindian ancestry, with Mexicans generally having the highest proportion of Amerindian ancestry, and the Caribbean groups (Cuban, Dominican, and Puerto Rican) have high African ancestry, with Dominicans generally having the largest proportion of African ancestry. In contrast, Cubans generally had the lowest proportion of Amerindian ancestry, and Mexicans the lowest proportion of African ancestry. Distributions of admixture proportions, as well as PC plots and additional information about the construction of the groupings are provided in [20].

Local ancestry inference has been reported in Browning et al. (2016) [23]. It was performed using RFMix [24] based on the genotyping values and a reference panel derived from the Human Genome Diversity Project [25] (HGDP) and the 1000 Genome Project [26]. This resulted in 14,815 intervals, each spanning tens to hundreds of thousands base pairs. For each interval and each participant, we obtained the likely count of these intervals (0, 1 or 2) that were inherited from a European, African, or Amerindian ancestor.

### Admixture and association mapping

In admixture mapping, we tested the trait associations of LAI counts inherited from a specific ancestry (e.g. African) against a baseline, the other two ancestries. In association mapping, we tested the trait associations of counts of effect alleles against a baseline, the other allele. Comparison between the admixture and the association mapping estimand and power in the scenario of two ancestries and a single causal genotype in an LAI is provided in S1 File. Both approaches used linear mixed models (LMM) adjusted for sex, age, study center, sampling weights (to protect against potential selection bias due to the study sampling scheme), and five principal components of genetics representing ancestry (to protect against population stratification), with random effects corresponding to kinship, household, and block unit. All analyses used the GENESIS R package [27]. The GWAS results stratified by genetic analysis groups are reported in a separate manuscript [10].

Our primary analysis was admixture mapping with secondary association testing employed to fine map specific association loci. For statistical significance of admixture mapping we used the *p*-value threshold 5.68 × 10^−5^ reported by Browning et al. (2016) [28] for the HCHS/SOL data set. However the multiple testing burden however is higher, due to the number of traits (4 traits, estimated as 2 independent traits using the simpleM method [29] based on the correlation between them) and of ancestries (3). Therefore, discovery of an association at genome-wide significance does not suffice and replication is needed for validating discoveries.

### Fine mapping of admixture mapping signal via association testing

After discovering genome-wide significant LAI associations (at the admixture mapping level), we attempted to fine-map these intervals to detect genotype associations that account for the signals. To determine whether a genotype association explains an admixture mapping signal, it suffices to use the genotype count as a covariate in the same regression analysis as the local ancestry count, to see if the local ancestry association becomes less statistically significant.

Each local ancestry interval corresponds to possibly thousands of imputed and hundreds of genotyped variants in the HCHS/SOL data set, many may not appear to be significantly associated with the trait (e.g. *p*-value> 10^−7^). Based on the power comparison provided in S1 File, admixture mapping may be much more powerful than association mapping when the allele frequency of the causal variant substantially differs between the compared ancestries. Therefore, we searched for genotype association loci by filtering variants in the interval according to both their *p*-value threshold and sometimes differences in effect allele frequencies (EAFs) between the two genetic analysis groups with the highest and lowest proportions of the ancestry of interest. Thus, if an admixture mapping association was discovered in a specific interval comparing the counts of Amerindian ancestry to other ancestries, we considered the EAF (which is easily calculated) of the Mexican and Cuban genetic analysis groups for variants in the interval. This is a practical alternative to calculating the more interesting quantity, the difference between EAFs in the ancestral populations, for all variants in the LAI, because it is computationally too intensive.

For relatively high *p*-values in association testing (e.g. ∼ 0.001) we required a difference in EAFs between the Mexican and the Cubans. For relatively low *p*-values (e.g. ∼ 10^−6^), we did not make any such restriction, because (1) the number of such variants is low, so stronger filtering is not required, and (2) admixture association may be detected by differences in the variant effect between the ancestral groups, even when the EAF is the same. In practice, we took a step-wise approach in which we relaxed the required *p*-value by factors of 10 (10^−6^, …, 10^−2^), where for low *p*-values (10^−3^, 10^−2^) we filtered by decreasing differences in EAFs (0.2, 0.15, 0.1, 0.5) of the two relevant genetic analysis groups. Finally, when searching for variants in the association analysis results, we utilized the heterogeneity of the HCHS/SOL cohort again by searching in both the results from the meta-analysis of all genetic analysis groups, the meta-analysis of only Caribbean, and the meta-analysis of only the Mainland genetic analysis groups reported in [10]. This was done because differences in the causal variant’s effect sizes between ancestral populations can cause an admixture mapping association, and in such settings, a variant association may be observed in only one of the Mainland/Caribbean groups due to the differences in admixture proportions between them.

Upon generating a list of potential variants associated with the trait, we pruned them to obtain lead variants (variants with lowest *p*-value in association testing) from each set of correlated variants, defined as the set of variants such that each variant has Pearson correlation of at most 0.4 with at least one other member of the set. We then applied the admixture mapping model while adjusting for these variants in the mixed model (conditional model), and also calculated the ancestry-specific EAF of these variants using ASAFE software. [30]

### Replication testing

We used a study of Southwest American Indians [31, 32] to validate associations of four SNPs, which are polymorphic in Amerindian ancestry, identified under admixture signals. The Pima Indian data had been collected as part of a population-based longitudinal study of health. Participants underwent biennial research examinations beginning at age ≥5 years that included measurements of blood pressure, height and weight to calculate BMI, and administration of a 75-g oral glucose tolerance test (OGTT). Informed consent was obtained from all subjects and ethics approval was received from the National Institute of Diabetes and Digestive and Kidney Diseases Institutional Review Board.

This study sample contained 2,347 full-heritage Pima Indians. Analysis was performed using a linear mixed model accounting for relatedness, and adjusted for age, sex, birth year, and the first 5 principal components. We consider an association significant if its one-sided *p*-value in the replication study is smaller than the Bonferroni threshold of 0.05/6 = 0.008.

## Results

### Sample characteristics

Table A in S1 File provides the sample characteristics in the combined sample, and in the Mainland (high proportion of Amerindian ancestry) and Caribbean (high proportion of African ancestry) sub-samples. While the distributions of age, sex, and Body Mass Index were similar across Mainland and Caribbean participants, hypertension was more prevalent in the Caribbeans, and accordingly, SBP, DBP, MAP, and PP were somewhat higher.

### Admixture mapping

Figs A-D in S1 File provides the Manhattan plots from all admixture mapping analyses for each of the investigated traits and from testing LAI counts of Amerindian, African, and European ancestries individually and in secondary analyses of all ancestries jointly. In a joint test, the effects of the counts of two ancestries (e.g. African and Amerindian) are modeled using two variables in the statistical models, and are compared to a baseline (e.g. the two LAIs are of European ancestry), using the 2-degrees-of-freedom null hypothesis stating that both ancestry effects are null. There were multiple significant local ancestry association regions in the analyses of Amerindian ancestry versus other ancestries: an association region on chromosome 6 (6p21.31) detected in both the MAP and SBP analyses, an association region on chromosome 11 (11q13.4) in the MAP analysis, and a PP region on chromosome 17 (17q25.3). In addition, a different association region on chromosome 6 was detected in the MAP and DBP analyses of African ancestry versus others. There were no significant associations in the European ancestry versus others analyses, nor in the joint analyses.

Table 1 provides the most significant LAIs in each of the association regions and analyses in terms of interval coordinates, estimated admixture mapping effect size, frequency of the tested ancestry at the interval, and *p*-values. Note that although we provide effect sizes, these are not biologically meaningful, because they depend on the ancestry frequencies (see S1 File). Moreover, these intervals may represent a complex summary across multiple causal variants in the interval.

**Table 1:** Top admixture association results in significant association regions. The lead LAI provides the coordinates of the most significant local ancestry interval in the region. Ancestry freq provides the proportion of intervals inferred as inherited from the tested ancestry across the 12,116 individuals (24,232 chromosome). Effect size, SE and *p*-values were estimated based on the linear model for the effect of the local ancestry count on the trait.

Region	Trait	Tested ancestry	Chr	lead LAI	Ancestry freq	Effect size	SE	*p*-value
6p21.31	SBP	Amerindian	6	(33971226, 34143292)	0.26	−1.31	0.29	6.57E-06
6p21.31	MAP	Amerindian	6	(35923246, 36230135)	0.27	−0.92	0.21	8.14E-06
6p12.3	MAP	African	6	(45501552, 45527132)	0.15	−1.06	0.26	5.52E-05
6p12.3	DBP	African	6	(45501552, 45527132)	0.15	−0.96	0.23	3.19E-05
11q13.4	MAP	Amerindian	11	(71243244, 71294802)	0.32	−0.85	0.21	4.67E-05
17q25.3	PP	Amerindian	17	(78252770, 78392234)	0.30	0.79	0.19	2.29E-05

### Conditional analysis

We sought to identify specific variants that account for the admixture mapping associations. We considered all genotyped and imputed SNPs assessed in the BP GWAS [10] performed in HCHS/SOL that are located within regions surpassing genome-wide significance in the current admixture mapping analyses. We selected SNPs according to significance of BP trait associations and differentiation of effect allele frequencies (EAFs) in the genetic analysis groups with high and low proportion of the tested ancestry, as described in the methods section. We did not detect any genotypic markers explaining the admixture association region in the African ancestry versus others analysis: that is, according to the most lenient criterion, no genotypes within the interval had *p*-value< 10^−2^ and EAF difference of at least 5% between the Dominican and Mexican genetic analysis groups. However, we identified variants explaining the three remaining association regions in Amerindian MAP, SBP and PP analyses. This is illustrated in Figs E-G in S1 File, via Manhattan plots comparing the primary (unconditional) analyses to the conditional ones.

Table 2 provides information about variants explaining the association regions, discussed below.

**Table 2:** Selected variants in top admixture mapping association regions, and their association testing results in stratified BP GWAS (stratified by genetic analysis groups and followed by a step of meta-analysis across the groups). All admixture mapping hits here were in the analysis of Amerindian ancestry versus other ancestries. We provide the effect estimate (Effect) from both the admixture mapping (Ancestry effect), and association mapping, the frequency of the ancestry (Ancestry freq), and the frequencies of the variant in the Mexican (high Amerindian ancestry) and Cuban (low Amerindian ancestry) genetic analysis groups. Genomic positions are in human build 37. A1 is the effect (tested) allele, A2 is the other allele. Type refers to imputation: ‘g’ is genotyped, ‘i’ imputed. Info is a measure of imputation quality. ^*a*^ For SNP rs118163160, the association analysis results when restricted to the Mainland groups were effect = −2.85, SE = 0.69, *p*-value = 3.84E-05. *P*-value for heterogeneity of the effect across the genetic analysis groups was 0.001.

Trait	Chr	Ancestry effect	Ancestry freq	rsID	position	A1	A2	Type	Info	A1 freq Mexican	A1 freq Cuban	Effect	SE	*p*-value
MAP	6	−0.92	0.27	rs112311534	31341578	G	A	i	0.96	0.80	0.68	−0.72	0.18	5.40E-05
				rs138753323	32173599	T	C	i	0.98	0.99	0.99	−3.19	0.72	8.53E-06
				rs75432840	34110808	C	G	g	1	0.68	0.94	0.82	0.20	4.39E-05
				rs138977532	36349802	C	T	g	1	0.81	0.98	1.00	0.25	4.62E-05
SBP	6	−1.31	0.26	rs112311534	31341578	G	A	i	0.96	0.80	0.68	−1.01	0.25	6.60E-05
				rs138753323	32173599	T	C	i	0.98	0.99	0.99	−3.76	1.02	2.18E-04
				rs75432840	34110808	C	G	g	1	0.68	0.94	1.13	0.28	6.16E-05
				rs138977532	36349802	C	T	g	1	0.81	0.98	1.46	0.35	2.98E-05
MAP	11	−0.85	0.32	rs118163160	71118878	C	T	i	1	0.98	0.96	−0.53^*a*^	0.46^*a*^	2.47E-01^*a*^
				rs139139046	71163354	G	C	i	0.84	0.90	0.98	0.93	0.35	7.68E-03
				rs7950649	71221331	C	T	g	1	0.92	0.86	−0.66	0.25	8.46E-03
PP	17	0.79	0.30	rs72849841	78272294	C	T	g	1	0.93	0.87	0.71	0.24	3.37E-03
				rs8079586	78308785	A	G	i	1	0.57	0.35	0.48	0.15	1.08E-03

#### MAP and SBP association region on chromosome 6

**Fig 1:**
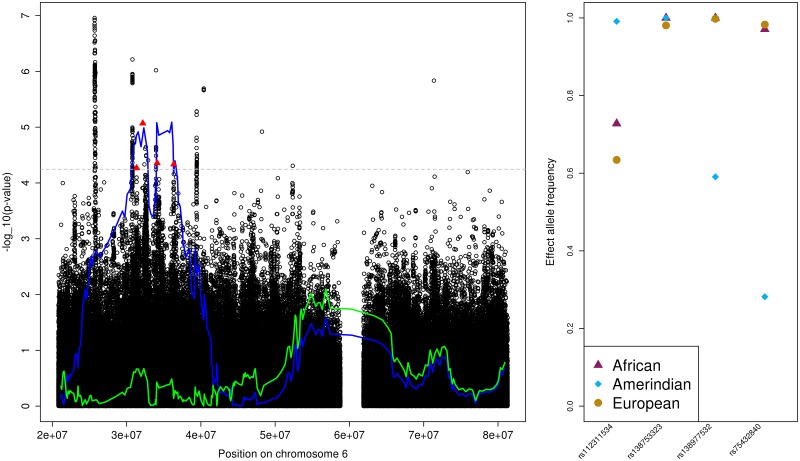
The MAP (and SBP) Amerindian admixture mapping region on chromosome 6. The left panel provides the admixture mapping results as two lines, with the blue line that crosses the genome-wide significance threshold (horizontal grey dashed line) representing results from the primary analysis and the other, green line, representing the results from the conditional analysis, and the association results in the same region as circles. Lines and points are given as -log(*p*-value, 10) against genomic positions. Filled triangles correspond to the SNPs used in the conditional analysis. The right panel provides the ancestry-specific effect allele frequencies (EAF) for each of the SNPs used in the conditional analysis, as estimated by ASAFE applied on the HCHS/SOL data set [30].

The 6p21.31 association region is quite large (multiple LAIs were statistically significant). There are multiple previously reported BP associations in this region: rs9266359, rs2021783 [5], rs805303 [3], rs2270860 [13], rs1563788 [11]. We first performed conditional admixture mapping analysis, conditioned on these variants. However, these variants did not explain the admixture association (Fig E in S1 File). We identified four variants that had significant *p*-values in association analysis, as described in the methods section. Three of the variants had large EAF differences between the Mexican and Cuban genetic analysis groups, while the most significant variant had roughly the same EAF across groups. We still adjusted for this variant, because the derivation in the S1 File shows that an admixture mapping association can be due to differences in effect sizes among ancestries. Fig 1 overlays admixture and association mapping results for the MAP Amerindian analysis, demonstrating that the variants rs112311534, rs138753323, rs75432840, and rs138977532, explain the admixture mapping association.

#### MAP association region on chromosome 11

We selected three variants based on association testing results and differences in EAFs between the Cuban and Mexican genetic analysis groups (Table 2). Interestingly one of these variants, namely rs118163160, had low a *p*-value among the Mainland groups only (*p*-value = 3.84E-05, compared to *p*-value = 0.28 in the combined cohort) but EAFs were similar across ancestries, suggesting that in this case there are differences in SNP effect sizes between the ancestries. These variants are in close proximity to rs1790370, previously reported by Wang et al. (2014) [33] in a candidate targeted association testing of BP traits with SNPs from vitamin-D pathway.

**Fig 2:**
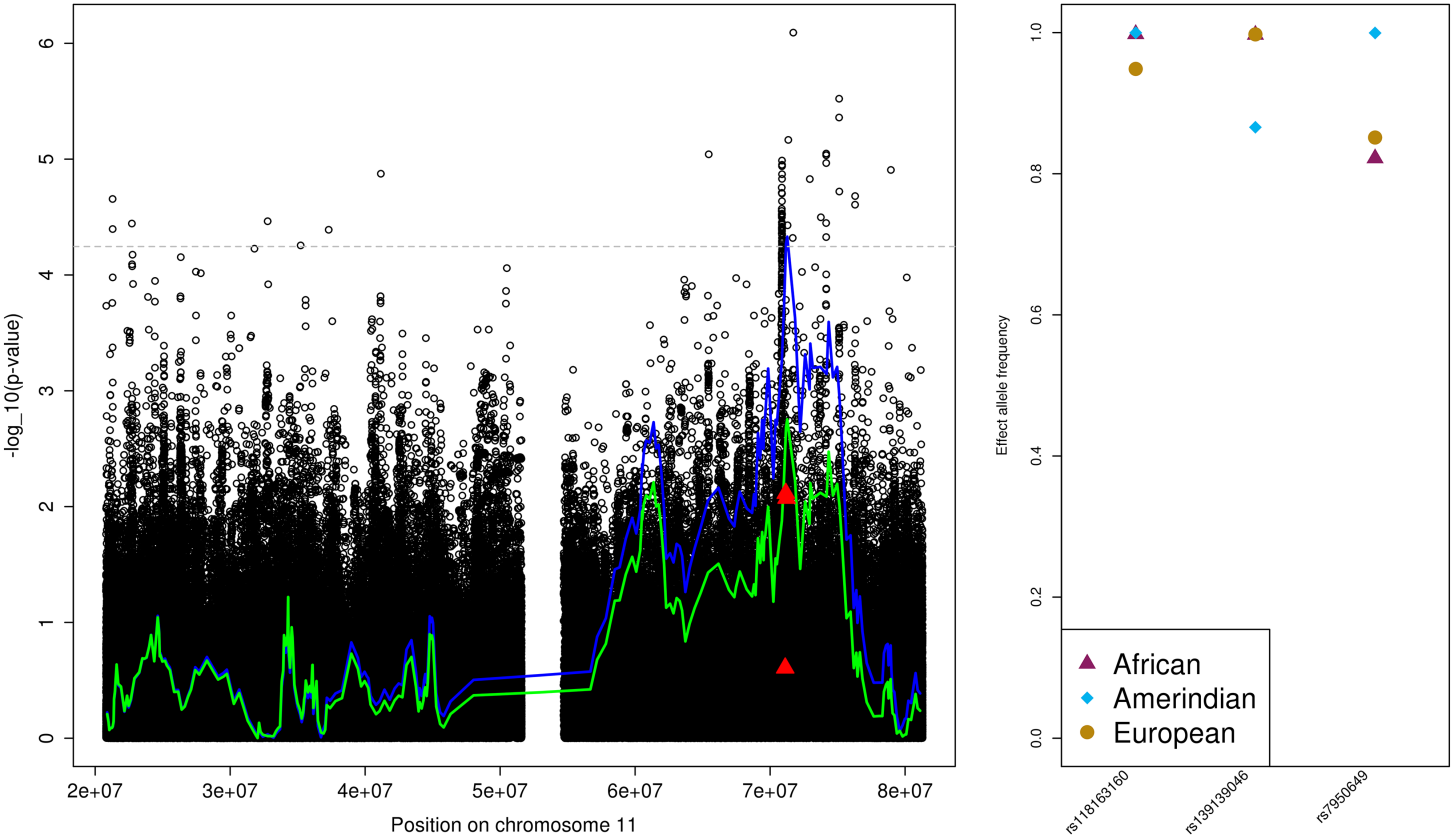
The MAP Amerindian admixture mapping region on chromosome 11. The left provides the admixture mapping results as two lines, with the blue line that crosses the genome-wide significance threshold (horizontal grey dashed line) representing results from the primary analysis and the other, green line, representing the results from the conditional analysis, and the association results in the same region as circles. Lines and points are given as -log(*p*-value, 10) against genomic positions. Filled triangles correspond to the SNPs used in the conditional analysis. The right panel provides the ancestry-specific effect allele frequencies (EAF) for each of the SNPs used in the conditional analysis, as estimated by ASAFE applied on the HCHS/SOL data set [30].

The selected variants partly explained the admixture peak, as could be seen in Fig 2. The *p*-value of the lead admixture mapping interval became 0.002 in the conditional analysis.

#### PP association region on chromosome 17

**Fig 3:**
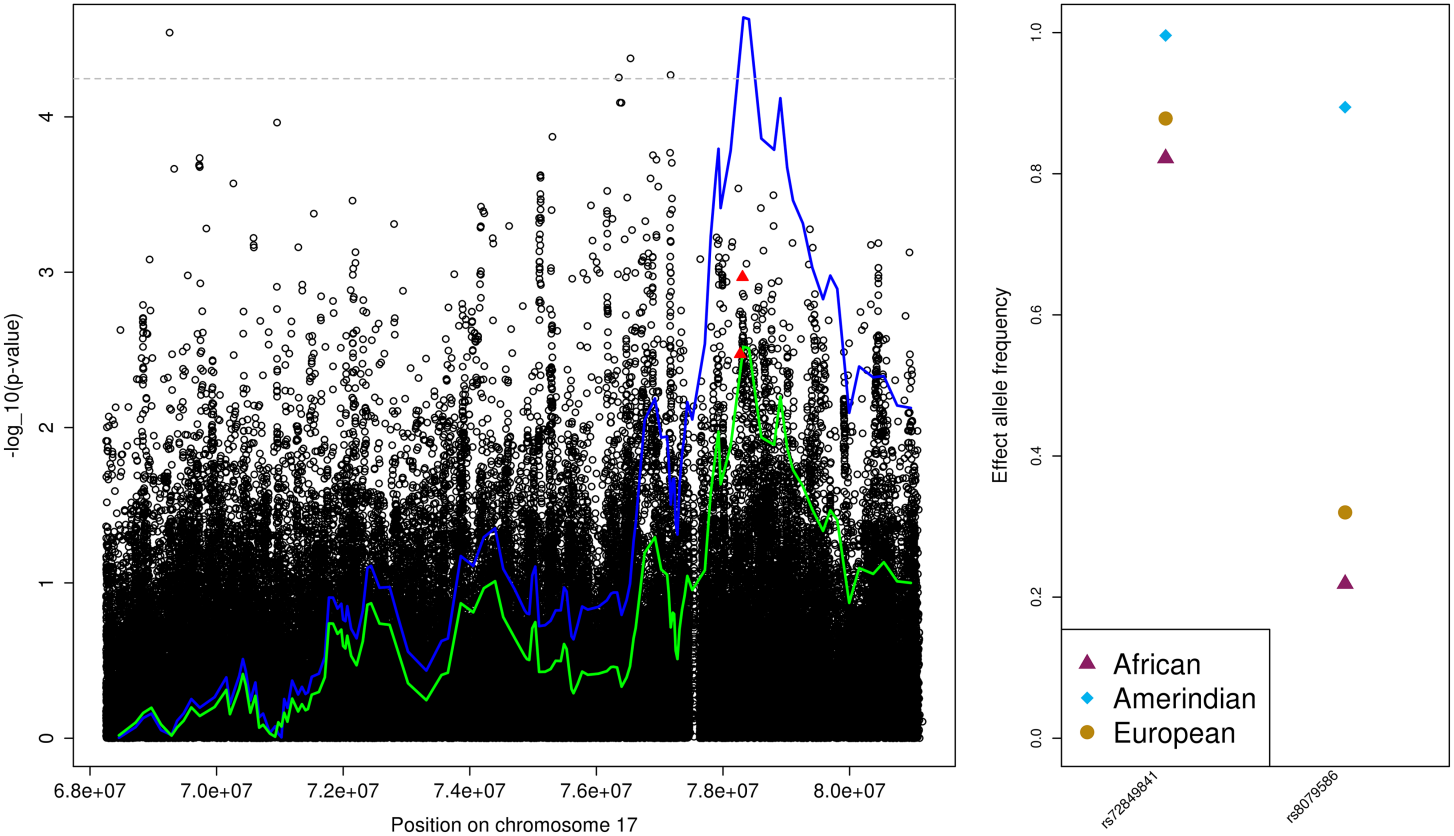
The PP Amerindian admixture mapping region on chromosome 17. The left panel provides the admixture mapping results as two lines, with the blue line that crosses the genome-wide significance threshold (horizontal grey dashed line) representing results from the primary analysis and the other, green line, representing the results from the conditional analysis, and the association results in the same region as circles. Lines and points are given as -log(*p*-value, 10) against genomic positions. Filled triangles correspond to the SNPs used in the conditional analysis. The right panel provides the ancestry-specific effect allele frequencies (EAF) for each of the SNPs used in the conditional analysis, as estimated by ASAFE applied on the HCHS/SOL data set [30].

There were no previously-reported BP loci in this region. We selected two variants with differentiated EAFs in the significant LAI in the 17q25.3 region. Their *p*-values were relatively high (0.001–0.004, see Table 2) but in the conditional analysis the admixture mapping *p*-value increased from 2.29E-05 in the primary analysis to 0.003, suggesting that these variants partially explain this signal. This is described in Fig 3.

### Replication analysis

We sought replication of the variants identified under the admixture mapping peaks on chromosomes 6, 11 and 17 in a study of Pima Indians (see methods). Four variants were polymorphic in Pima Indians and genotypic data was available to assess replication. Table 3 compares the association analysis results for these variants in the HCHS/SOL and in the replication study. None of the variant associations replicate when accounting for multiple testing, though rs138977532 had a one-sided *p*-value of 0.02 in association testing of MAP and 0.04 in testing SBP. The estimated Amerindian frequencies and the frequency in Pima Indians varied substantially, with one variant (rs72849841) estimated as almost monomorphic in the HCHS/SOL Amerindian ancestry (MAF = 0.004), while being quite polymorphic in Pima Indians (MAF = 0.06). The frequency of this variants in the 1000 Genome AMR population was 0.07, as in the Pima Indians.

**Table 3:** Results from replication testing of SNPs in significant admixture association regions in 2,347 Pima Indians. Genomic positions are in human build 37. A1 is the effect (tested) allele, A2 is the other allele. For each of HCHS/SOL and the replication study, we provide allele frequencies, estimated effect sizes, and *p*-values. For the replication study, *p*-values are one-sided as determined by the direction of association observed in the HCHS/SOL. For the HCHS/SOL frequencies we report “Amer freq”: the estimated allele frequency in the Amerindian ancestry in the HCHS/SOL, as estimated using ASAFE [30].

Trait	Chr	rsID	position	A1	A2	HCHS/SOL			Pima Indians		
						Amer freq	Effect	*p*-value	freq	Effect	*p*-value (one-sided)
MAP	6	rs75432840	34110808	C	G	0.28	0.82	4.39E-05	0.43	−0.26	0.77
MAP	6	rs138977532	36349802	C	T	0.59	1	4.62E-05	0.63	0.74	0.02
SBP	6	rs75432840	34110808	C	G	0.28	1.13	6.16E-05	0.43	−0.75	0.92
SBP	6	rs138977532	36349802	C	T	0.59	1.46	2.98E-05	0.63	0.89	0.04
MAP	11	rs139139046	71163354	G	C	0.87	0.93	7.68E-03	0.71	0.15	0.35
PP	17	rs72849841	78272294	C	T	0.996	0.71	3.37E-03	0.94	−1.43	0.95

## Discussion

We performed admixture mapping of BP traits in a heterogeneous sample of US Hispanics/Latinos with the intent of identifying novel BP loci. In admixture mapping, counts of local ancestry at intervals are tested for association with a trait, and this approach has the potential to be more powerful than association analysis to identify variants with large EAF differences across ancestral populations (see S1 File for power comparison in the simple case of two ancestral populations). We show that admixture mapping association can also be detected when effect sizes differ between ancestral populations. However, identified intervals may encompass a large number of variants, both common and rare, that can drive the association with the trait. Therefore unlike association testing, in which a specific variant association is estimated and tested, in tested local ancestry intervals the “causal” variant(s) accounting for the association may be one or more untyped variants, which may not have an available proxy even with imputed data.

In this admixture mapping study, we detected four associated regions in which local ancestry was associated with quantitative BP traits. We utilized the pre-calculated allele frequencies and the HCHS/SOL genetic analysis groups, in conjunction with information about the ancestry proportions in each group, to follow up on associated LAIs and potentially fine-map specific association loci. We also considered GWAS analysis results in Mainland (Mexican, Central, and South American) and Caribbean (Cuban, Dominican, and Puerto Rican) Hispanic/Latino groups that have different distributions of ancestry proportion, because admixture mapping association can be caused by different effect sizes between ancestries. For one region (where local African ancestry was specifically associated with BP), we did not find any candidate variants. For the other three associated regions (specifically associated with local Amerindian ancestry), we proposed a few variants explaining the admixture mapping association. Four proposed variants in the 6p21.31 region completely explained the local Amerindian ancestry association. These variants may tag an associated Amerindian haplotype in the region. In the 11q13.4 MAP Amerindian ancestry association region, one of the candidate variants was associated with MAP only in the Mainland-specific analysis reported in [10], consistent with a hypothesis that this variant association have different effect size in the Amerindian ancestral population compared to the other ancestral populations. Unfortunately, this variant was not available in our replication cohort.

Because the identified admixture mapping signals were identified in analyses comparing Amerindian ancestry to other ancestries, we tested the association with BP traits in a study of Pima Indians but these associations did not replicate when accounting for multiple testing. However, one variant in the chromosome 6 association region had replication one-sided *p*-values of 0.02 and 0.04 with MAP and SBP, respectively. One-sided testing, where the side was based on the direction of association in the HCHS/SOL, has been shown to be more powerful while still providing better protection from type 1 error than two-sided testing in the replication stage. [34] Possible reasons for lack of replication are (1) lack of power for replication given the small sample size of the replication study, (2) the variants may not be strong proxies of the true causal variants, (3) gene environment interaction, e.g. the effect size may be different in the replication population, (4) the causal variants may be outside the boundaries of the most significant local ancestry intervals, as there is an uncertainty inferring local ancestries, and finally (5) the associations may be false positives. Admixture mapping analyses performed in additional samples of Hispanics/Latinos may help resolve these issues.

Fig 2 shows that there are multiple variants near the chromosome 11 admixture peak that have higher significance in the single variant association testing. To assess whether these variants better explain the admixture mapping peak, we performed a single conditional analysis with the most significant common SNP in the region. The admixture mapping peak became slightly less significant, with the p-value of the lead LAI becoming 5.36E-04. Though we cannot make any definite conclusions about the tested variant contributing to the local ancestry association, we decided not to pursue fine mapping using variants outside the significant LAIs, due to the lack of appropriate rules for considering such variants. More generally, the ad-hoc approach that we pursued here for selecting SNP candidates should be improved upon in the future and more precise criteria for determining whether a variant or a set of variants explained an admixture mapping association are needed. A potential avenue for such improvement is the joint testing of local ancestry counts and of (possibly multiple) genotypes. Such tests have been previously proposed [35] but not in the context of mixed model. Such an approach may be useful for fine mapping following admixture mapping, or as a primary GWAS analysis. Most promising, however, are fine mapping strategies via whole-genome sequencing studies, once they are available in Hispanic/Latino and American Indians populations.

In summary, we detected multiple regions of associations with local ancestry for BP traits. These loci are distinct from genomic regions reported in our recent report of BP GWAS in the HCHS/SOL. Such associations are driven by genetic variants at the local ancestry interval. We provided a systematic approach to identify the underlying variants at each region. However, these variants may not be well tagged by genotyped or imputed variants in the data set. While we were able to detect a few candidate variants driving the Amerindian association regions, and one association had p-value = 0.02 in the replication study, none of the associations replicated after accounting for multiple testing in a small sample of Pima Indians. Unfortunately, we did not have access to any larger or additional sample of American Indians for further replication. We did not detect any variants driving the African Ancestry association region, suggesting that current genotyping and imputation platforms are lacking in coverage for less minority populations or that rare variants drive these associations. Whole-genome sequencing studies in Hispanics/Latinos, American Indians, and African Americans are needed for additional and more complete fine mapping efforts of complex traits.

## Supporting information

S1 File The supplementary information contains a tables with HCHS/SOL cohort characteristics, Manhattan plots describing the results of primary and conditional analyses, a mathematical derivation explaining admixture mapping under minor allele frequencies and different effect sizes between ancestries, and its comparison to association mapping (i.e. testing local ancestry counts versus testing genotypes counts), and finally, power comparisons between the two approaches. (PDF)
