# Supplementary material for: Admixture mapping in the Hispanic Community Health Study/Study of Latinos reveals regions of genetic associations with blood pressure traits
Source: PLoS One. 2017 Nov 20;12(11):e0188400. doi: 10.1371/journal.pone.0188400 (PMC5695820; doi:10.1371/journal.pone.0188400)
Supplement: S1 File — (PDF) [file pone.0188400.s001.pdf]

Supplementary Information
Admixture mapping in the Hispanic Community Health Study/Study of Latinos reveals regions of genetic associations with blood pressure traits

Tamar Sofer[1,2*], Leslie J. Baier[3], Sharon R. Browning[4], Timothy A. Thornton[4], Gregory A. Talavera[5], Sylvia Wassertheil-Smoller[6], Martha L. Daviglus[7,8], Robert Hanson[3], Sayuko Kobes[3], Richard S. Cooper[9], Jianwen Cai[10], Daniel Levy[11,12], Alex P. Reiner[13], Nora Franceschini[14]

[1] Division of Sleep and Circadian Disorders, Department of Sleep Medicine, Brigham and Women's Hospital, Boston, MA, USA;
[2] Department of Medicine, Harvard Medical School, Boston, MA, USA;
[3] Phoenix Epidemiology and Clinical Research Branch, NIDDK, NIH, Phoenix, AZ, USA;
[4] Department of Biostatistics, University of Washington, Seattle, WA, USA;
[5] Division of Health Promotion and Behavioral Science, San Diego State University, San Diego, CA, USA;
[6] Department of Epidemiology and Population Health, Albert Einstein College of Medicine, Bronx, NY, USA;
[7] Feinberg School of Medicine, Northwestern University, Chicago, IL, USA;
[8] Institute for Minority Health Research, University of Illinois at Chicago, Chicago, IL, USA;
[9] Department of Public Health Sciences, Stritch School of Medicine, Loyola University Chicago, Chicago, IL, USA;
[10] Department of Biostatistics, University of North Carolina, Chapel Hill, NC, USA;
[11] The Framingham Heart Study, Framingham, MA, USA;
[12] Population Sciences Branch, National Heart, Lung, and Blood Institute, National Institutes of Health, Bethesda, MD, USA;
[13] Division of Public Health Sciences, Fred Hutchinson Cancer Research Center, Seattle,WA, USA;
[14] Department of Epidemiology, University of North Carolina, Chapel Hill, NC, USA.

Correspondence:
Tamar Sofer, PhD
E-mail: tsofer@bwh.harvard.edu

Contents

1 HCHS/SOL cohort characteristics **2**

2 Manhattan plots from primary (non-conditional) admixture mapping analyses **4**

3 Manhattan plots comparing primary to conditional analyses **9**

4 Comparing admixture and association mapping **11**
4.1 The linear model local ancestries 11
4.2 The admixture mapping estimand 12

4.2.1 When the variant effect is the same in the two populations 12
4.2.2 When the variant effect differ between the two populations 12

5 Comparing power between association analysis and admixture mapping 13
5.1 Additional power considerations 13

1 HCHS/SOL cohort characteristics

	Overall	Mainland	Caribbean	CentralAmerican	SouthAmerican	Mexican	PuertoRican	Cuban	Dominican
n	12116	6632	5484	1334	878	4420	2151	2197	1136
age	46 (14)	45 (14)	48 (14)	45 (13)	47 (13)	45 (14)	48 (14)	49 (13)	45 (14)
female sex	7147 (59)%	3992 (60.2)%	3155 (57.5)%	788 (59.1)%	524 (59.7)%	2680 (60.6)%	1249 (58.1)%	1164 (53)%	742 (65.3)%
BMI	30 (6)	30 (5.8)	30 (6.3)	30 (5.8)	29 (5.2)	30 (5.9)	31 (6.9)	29 (5.8)	29 (5.7)
Hypertension	3411 (28.2)%	1458 (22)%	1953 (35.6)%	339 (25.4)%	178 (20.3)%	941 (21.3)%	797 (37.1)%	780 (35.5)%	376 (33.1)%
SBP	125 (20.2)	122 (19.1)	128 (20.8)	125 (20.3)	122 (19.9)	121 (18.5)	128 (21.1)	128 (20.4)	128 (21)
DBP	75 (11.9)	73 (11.2)	78 (12.1)	75 (11.5)	72 (11.6)	72 (11)	77 (12.5)	78 (11.8)	78 (12.1)
MAP	92 (13.8)	89 (13)	95 (14.1)	92 (13.6)	89 (13.6)	89 (12.7)	94 (14.4)	95 (13.7)	95 (14.2)
PP	50 (13.4)	49 (12.8)	51 (14.1)	49 (13.7)	49 (13.2)	49 (12.4)	51 (14.3)	50 (14.1)	50 (13.8)

Table A: Characteristics of HCHS/SOL study participants 2008-2011, overall, in Mainland and Caribbean groups, and in the individual background groups (also called genetic analysis groups). Means and standard deviations (SD) of the continuous BP traits were calculated after adjustment for using hypertensive medication.

2 Manhattan plots from primary (non-conditional) admixture mapping analyses

(a) Amerindian ancestry vs others

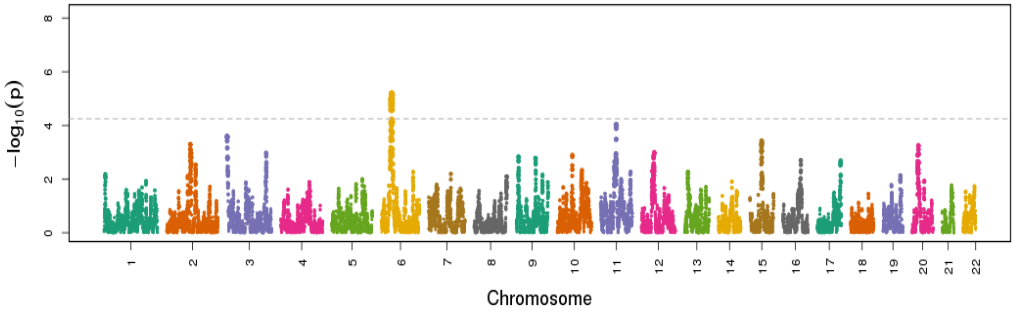

(b) African ancestry versus others

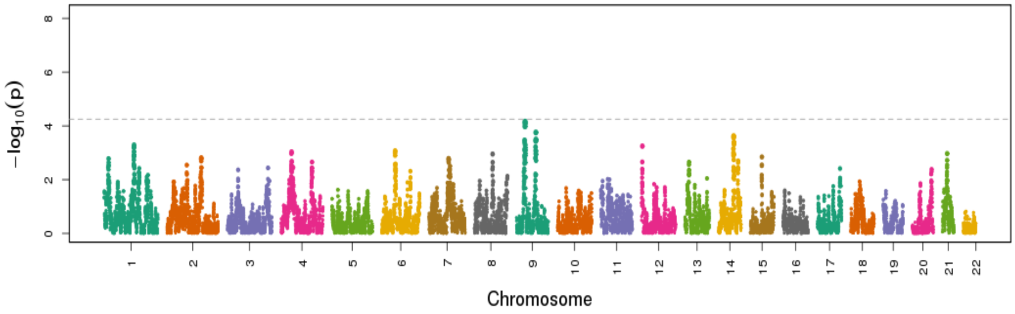

(c) European ancestry vs others

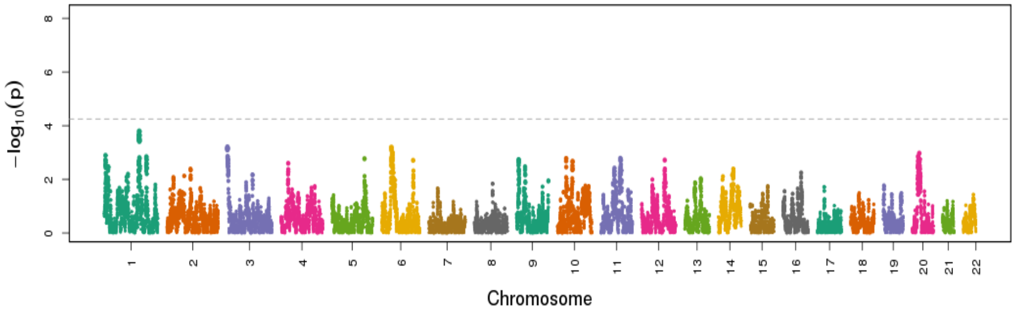

(d) Joint test of all ancestries

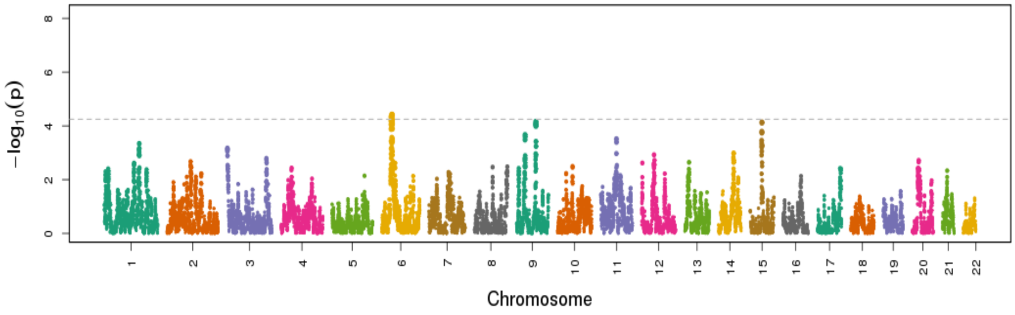

Figure A: **Manhattan plots from admixture mapping of SBP.**

(a) Amerindian ancestry vs others

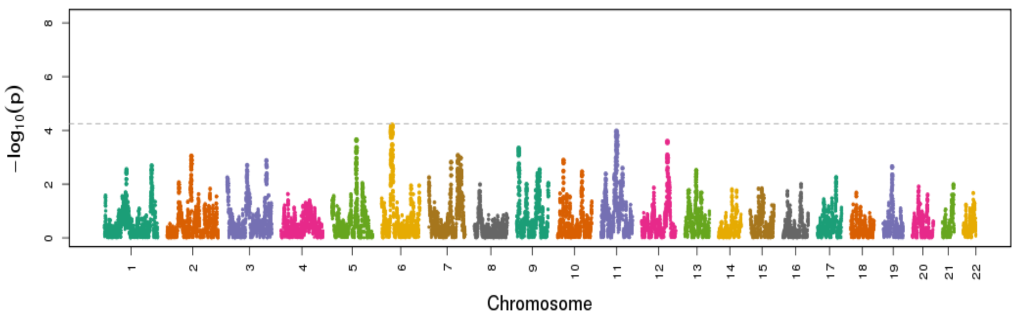

(b) African ancestry versus others

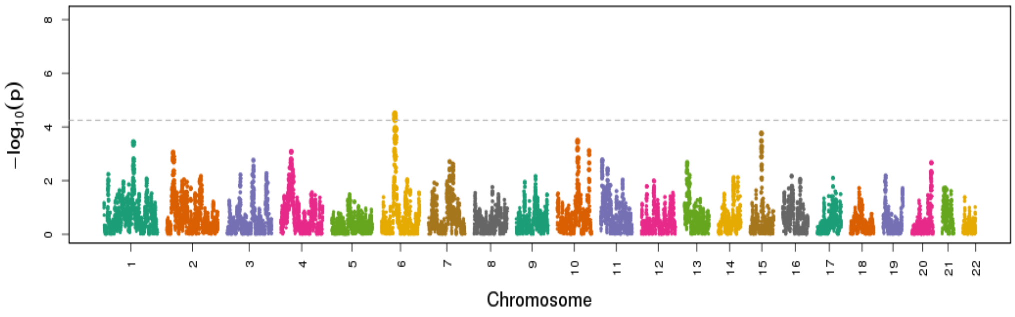

(c) European ancestry vs others

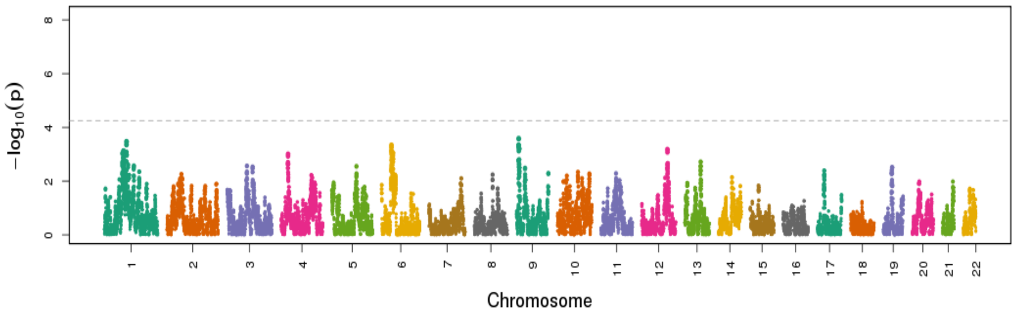

(d) Joint test of all ancestries

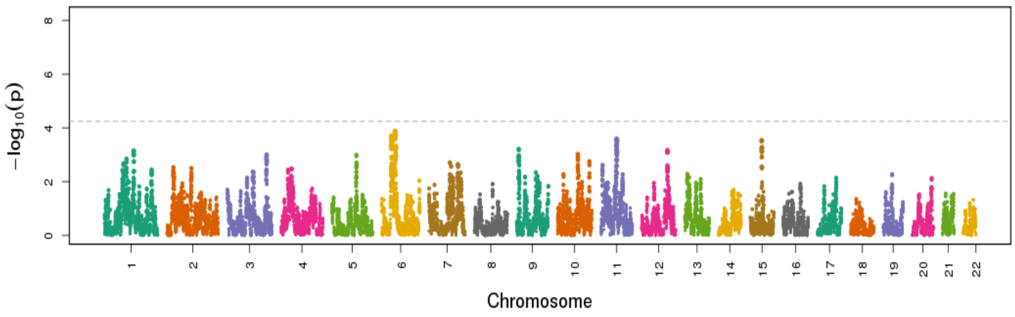

Figure B: **Manhattan plots from admixture mapping of DBP.**

(a) Amerindian ancestry vs others

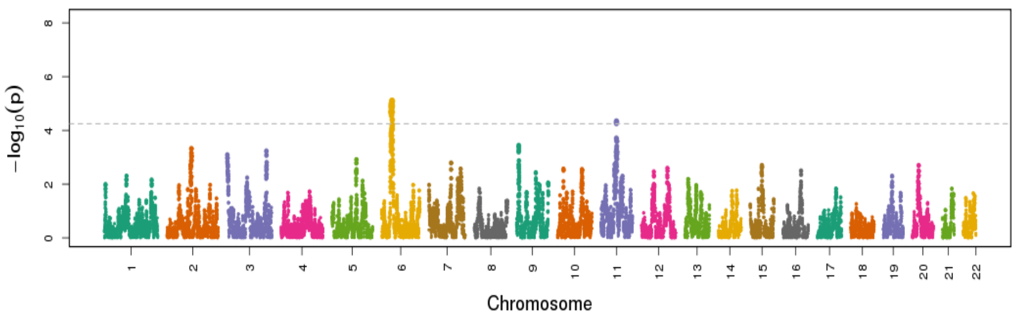

(b) African ancestry versus others

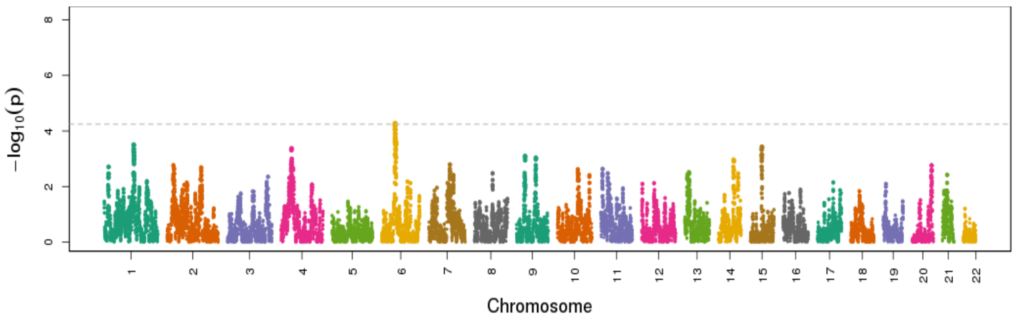

(c) European ancestry vs others

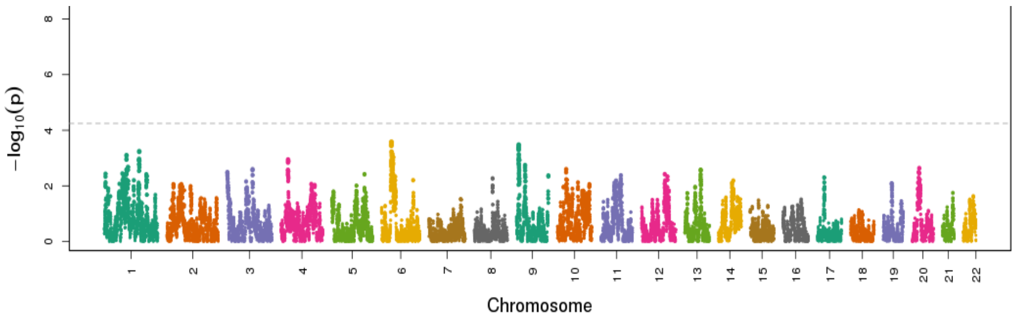

(d) Joint test of all ancestries

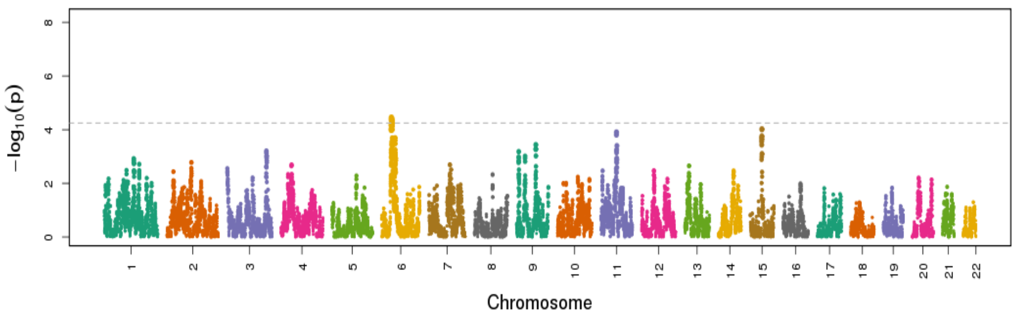

Figure C: **Manhattan plots from admixture mapping of MAP.**

(a) Amerindian ancestry vs others

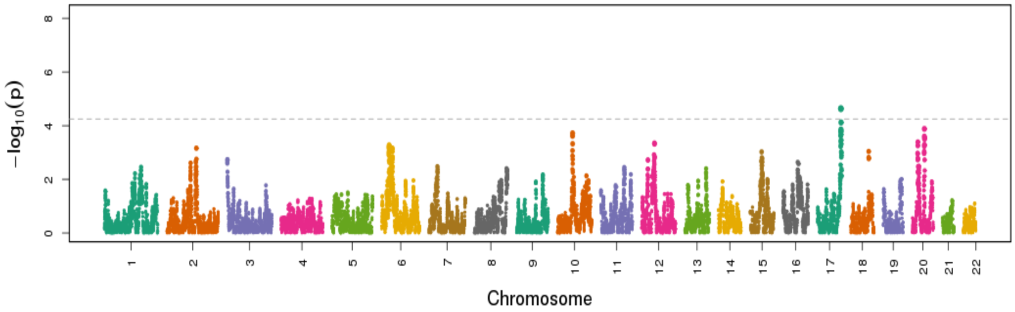

(b) African ancestry versus others

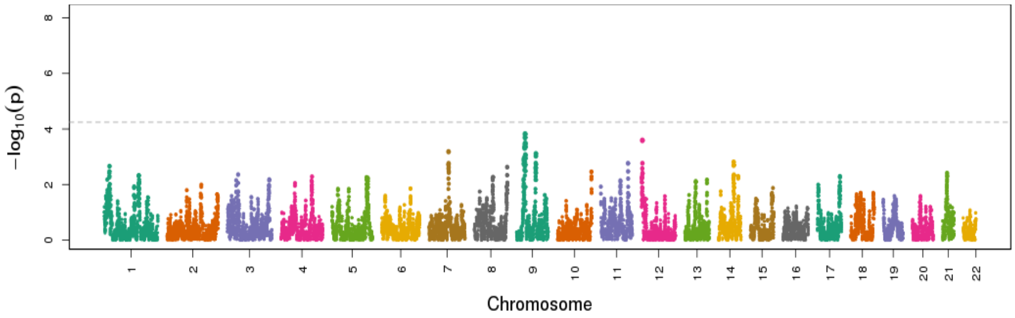

(c) European ancestry vs others

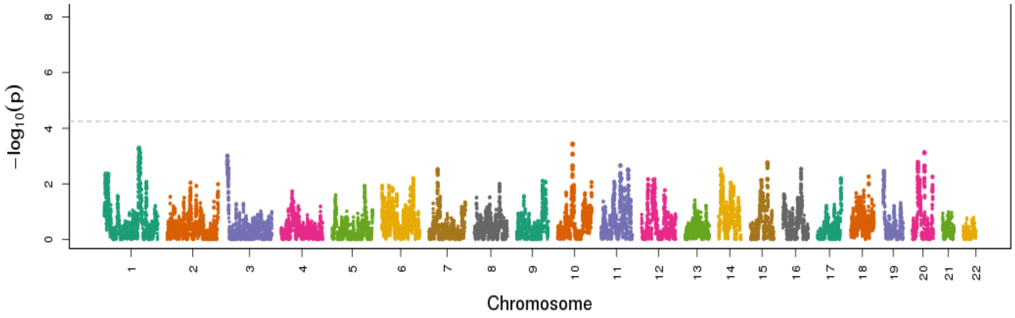

(d) Joint test of all ancestries

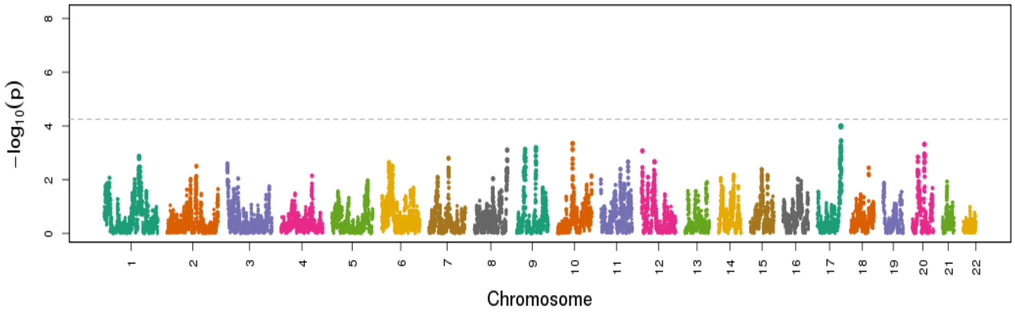

Figure D: **Manhattan plots from admixture mapping of PP.**

3 Manhattan plots comparing primary to conditional analyses

(a) With no conditioning

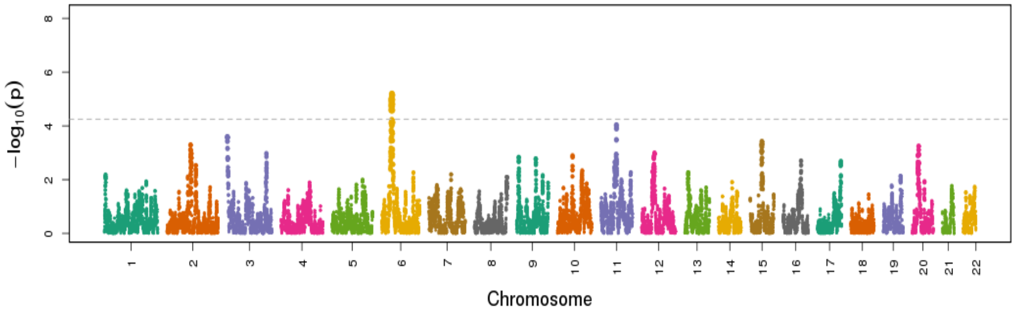

(b) Conditional on known association SNPs

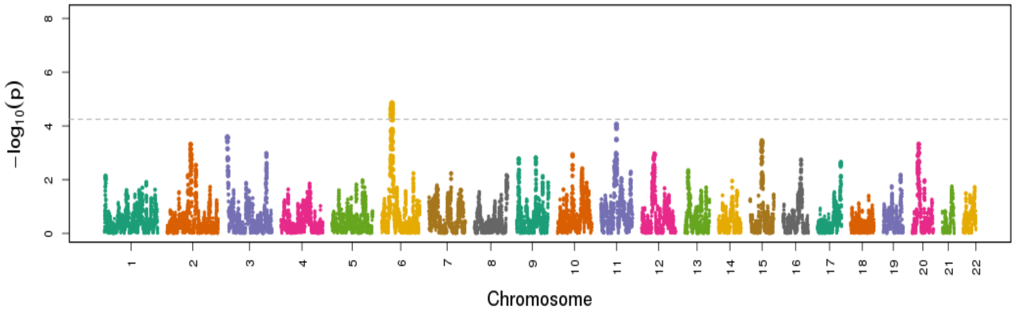

(c) Conditional on SNPs selected based on significance level and differentiation of frequencies

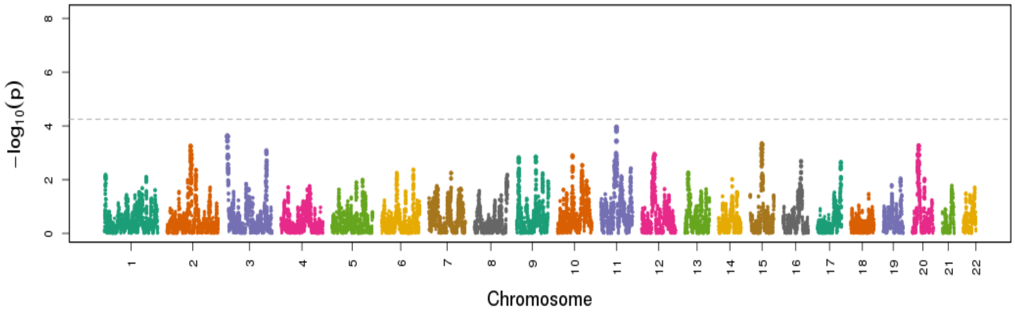

Figure E: Manhattan plots from admixture mapping of SBP (Amerindian ancestry vs others), focusing on the local ancestry association region on chromosome 6.

(a) With no conditioning

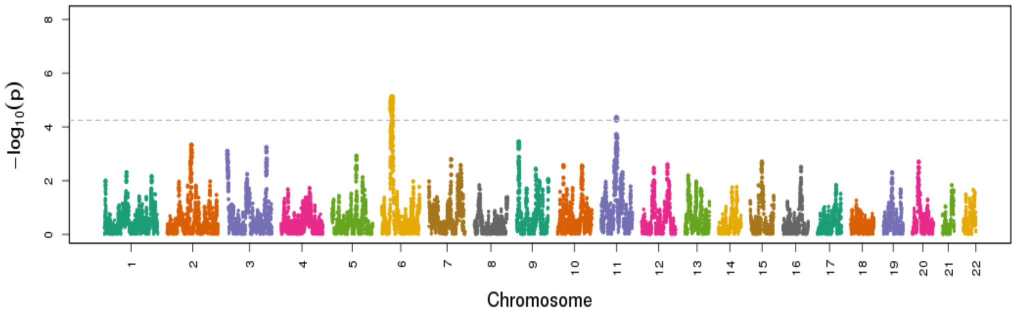

(b) Conditional on from chromosome 11 identified by significance level and differentiation of frequencies

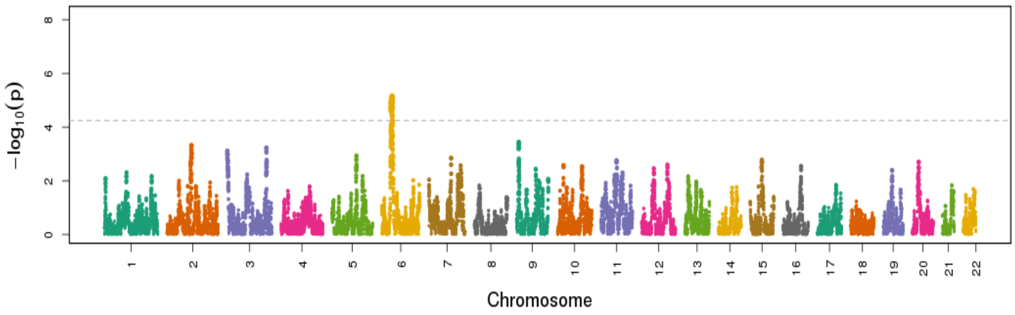

(c) Conditional on from chromosome 6 identified by significance level and differentiation of frequencies

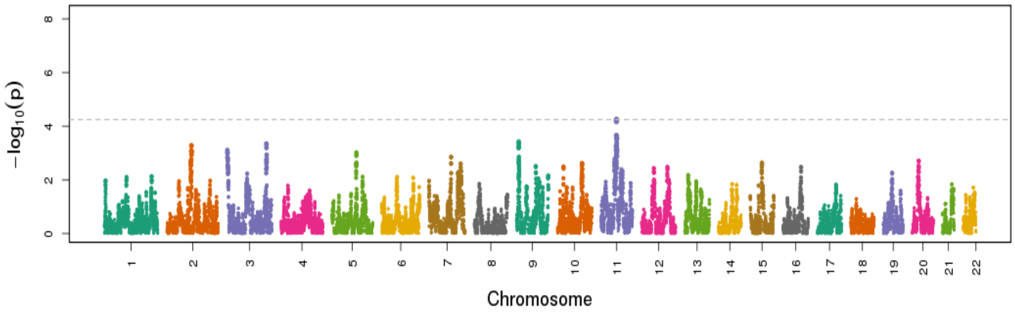

Figure F: Manhattan plots from admixture mapping of MAP (Amerindian ancestry vs others), focusing on the local ancestry association regions on chromosome 6 and chromosome 11.

(a) With no conditioning

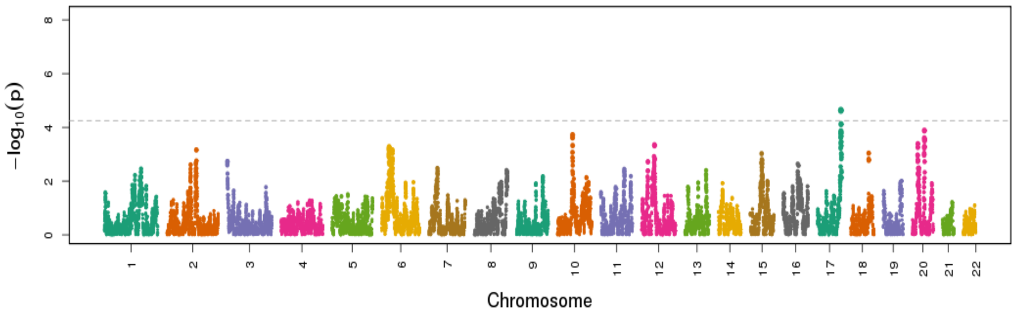

(b) Conditional on from chromosome 17 identified by significance level and differentiation of frequencies

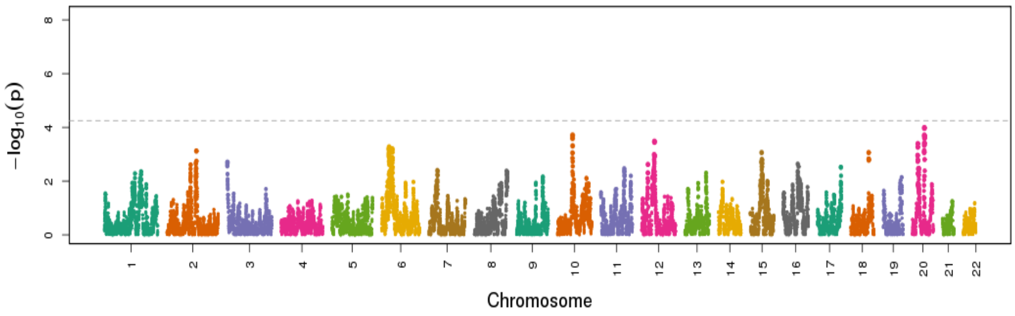

Figure G: Manhattan plots from admixture mapping of PP (Amerindian ancestry vs others), focusing on the local ancestry association region on chromosome 17.

4 Comparing admixture and association mapping

4.1 The linear model local ancestries

Suppose that an outcome y follows the linear model

$$y_i = \alpha_0 + \boldsymbol{x}_i^T \boldsymbol{\alpha} + g_i \beta + \epsilon_i, i = 1, \dots, n$$

where $\boldsymbol{\alpha}$ are covariate effects, $\boldsymbol{x}_i$ is a vector of covariates for participants i, g_i is the genotype count (or dosage) of participants i, β its effect, and ϵ_i is a random error, independent of covariates and genotypes effects (but not necessarily independent between participants). Note that we assume here that the genotype effect is the same for all individuals, regardless of subgrouping, e.g. local genetic ancestry. Suppose further that study individuals are admixed, and for simplicity, between two parental population. Therefore, variant g could have been inherited by descent from ancestry A_1 or from ancestry A_2. Assuming that there are intervals with known local ancestry (LAI, local ancestry intervals), in each such interval all participant genotypes on each chromosome are assumed to originate from the same ancestry.

Focusing on a given local ancestry inferred interval, each individual i has two genotype alleles inherited from genotype g (for all genotypes in the interval; here we focus on a single genotype). Genotype g can take the values a or b. In a model in which allele a is the effect allele, we say that $g_{i1} = 0$ if the allele of g on one of the chromosomes is b and $g_{i1} = 1$ if it is a. Similarly, g_{i2} can get the values 0 or 1. More formally, we have that:

$$g_{ij} = \begin{cases} 0 & \text{if genotype } g \text{ on copy } j \text{ its chromosome} = b \\ 1 & \text{if genotype } g \text{ on copy } j \text{ its chromosome} = a \end{cases}$$

Since g_{ij} may be either of A_1 or A_2, it has the conditional expected values:

$$E[g_{ij} = 1|A_k] = Pr\left(g_{ij} = 1|\text{LAI on copy } j \text{ of chromosome from population } k\right) = p_k$$

which is the a allele frequency in population $A_k, k = 1, 2$.

4.2 The admixture mapping estimand

4.2.1 When the variant effect is the same in the two populations

In admixture mapping, we regress the outcomes against counts of (estimated) local ancestries. Therefore, our working model is

$$y_i = \alpha_0 + \boldsymbol{x}_i^T\boldsymbol{\alpha} + a_{i1}\gamma + a_{i2}\gamma + \epsilon_i, i = 1, \dots, n$$

where

$$a_{ij} = \begin{cases} 0 & \text{if the ancestry of LAI of genotype } g \text{ on copy } j \text{ of its chromosome} = A_1 \\ 1 & \text{if the ancestry of LAI of genotype } g \text{ on copy } j \text{ its chromosome} = A_2 \end{cases}$$

Note that genotype g is not directly modeled, so that the model and the estimated effect γ does not explicitly depend on genotypes in the LAI. However the relationship between the admixture mapping estimand and the genotype effect estimand can be studied, specifically under the single allelic model which assumes that only a single genotype in the interval is associated with the outcome.

To characterize the estimand of the admixture mapping γ, consider the expected value of the outcome under various settings of local ancestry:

$$\begin{aligned} E[y_i|a_{i1}=0,a_{i2}=0] &= \alpha_0 + \boldsymbol{x}_i^T\boldsymbol{\alpha} + \gamma Pr[g_{i1}=1|g_{i1} \text{ from ancestry } A_1] \\ &\quad + \gamma Pr[g_{i2}=1|g_{i2} \text{ from ancestry } A_1] = \alpha_0 + \boldsymbol{x}_i^T\boldsymbol{\alpha} + 2\beta p_1 \\ E[y_i|a_{i1}=1,a_{i2}=0] &= \alpha_0 + \boldsymbol{x}_i^T\boldsymbol{\alpha} + \beta Pr[g_{i1}=1|g_{i1} \text{ from ancestry } A_2] \\ &\quad + \beta Pr[g_{i2}=1|g_{i2} \text{ from ancestry } A_1] = \alpha_0 + \boldsymbol{x}_i^T\boldsymbol{\alpha} + \beta p_2 + \beta p_1 \\ E[y_i|a_{i1}=1,a_{i2}=0] &= \alpha_0 + \boldsymbol{x}_i^T\boldsymbol{\alpha} + \beta Pr[g_{i1}=1|g_{i1} \text{ from ancestry } A_1] \\ &\quad + \beta Pr[g_{i2}=1|g_{i2} \text{ from ancestry } A_2] = \alpha_0 + \boldsymbol{x}_i^T\boldsymbol{\alpha} + \beta p_1 + \beta p_2 \\ E[y_i|a_{i1}=1,a_{i2}=1] &= \alpha_0 + \boldsymbol{x}_i^T\boldsymbol{\alpha} + \beta Pr[g_i1=1|g_{i1} \text{ from ancestry } A_2] \\ &\quad + \beta Pr[g_{i2}=1|g_{i2} \text{ from ancestry } A_2] = \alpha_0 + \boldsymbol{x}_i^T\boldsymbol{\alpha} + 2\beta p_2 \end{aligned}$$

Thus, the effect of increasing the A_2 ancestry count by 1 is $\gamma = \beta(p_2 - p_1)$, depending on both the genotype effect and the difference between the allele frequencies of the two ancestries.

4.2.2 When the variant effect differ between the two populations

Assume now different effects between the two populations, say β_1 for A_1 in β_2 for A_2. We get:

$$\begin{aligned} E[y_i|a_{i1}=0,a_{i2}=0] &= \alpha_0 + \boldsymbol{x}_i^T\boldsymbol{\alpha} + \gamma Pr[g_{i1}=1|g_{i1} \text{ from ancestry } A_1] \\ &\quad + \gamma Pr[g_{i2}=1|g_{i2} \text{ from ancestry } A_1] = \alpha_0 + \boldsymbol{x}_i^T\boldsymbol{\alpha} + 2\beta_1 p_1 \\ E[y_i|a_{i1}=1,a_{i2}=0] &= \alpha_0 + \boldsymbol{x}_i^T\boldsymbol{\alpha} + \beta Pr[g_{i1}=1|g_{i1} \text{ from ancestry } A_2] \\ &\quad + \beta Pr[g_{i2}=1|g_{i2} \text{ from ancestry } A_1] = \alpha_0 + \boldsymbol{x}_i^T\boldsymbol{\alpha} + \beta_2 p_2 + \beta_1 p_1 \\ E[y_i|a_{i1}=1,a_{i2}=0] &= \alpha_0 + \boldsymbol{x}_i^T\boldsymbol{\alpha} + \beta Pr[g_{i1}=1|g_{i1} \text{ from ancestry } A_1] \\ &\quad + \beta Pr[g_{i2}=1|g_{i2} \text{ from ancestry } A_2] = \alpha_0 + \boldsymbol{x}_i^T\boldsymbol{\alpha} + \beta_1 p_1 + \beta_2 p_2 \\ E[y_i|a_{i1}=1,a_{i2}=1] &= \alpha_0 + \boldsymbol{x}_i^T\boldsymbol{\alpha} + \beta Pr[g_i1=1|g_{i1} \text{ from ancestry } A_2] \\ &\quad + \beta Pr[g_{i2}=1|g_{i2} \text{ from ancestry } A_2] = \alpha_0 + \boldsymbol{x}_i^T\boldsymbol{\alpha} + 2\beta_2 p_2, \end{aligned}$$

so that $\gamma = (\beta_2 p_2 - \beta_1 p_1)$. Here, the admixture mapping estimator may detect an effect even if $p_1 = p_2$, in contrast to the same effect scenario.

5 Comparing power between association analysis and admixture mapping

Power for testing variants depend on their frequency, effect size, sample size, and also, the p-value threshold used for the analysis. In the case of admixture mapping, the frequency in question is in fact the frequency of the one of the ancestries. While there are many variables to consider, we here demonstrate a few scenarios, to consider cases in which admixture mapping is more powerful than association testing, in the two ancestries scenarios and under the same effect size assumption. Our calculations are based on the accepted p-value threshold for association analysis, the so-called genome-wide significance threshold 5×10^{-8}, and the p-value threshold for admixture mapping found by Brown et al. (2017)[?] for the HCHS/SOL: 5.68×10^{-5}.

Note that another scenario of higher power for admixture mapping is that of non-genotyped/imputed variant in an interval with inferred local ancestry. These variants could not possibly be detected by association analysis, but admixture mapping may yield significant associations.

Denote the probability of a variant to be inherited from ancestry A_1 by p_a. An association analysis, that does not make use of local ancestry information, has that the frequency of the genotype allele is

$$\begin{aligned} p(g=1) &= p(g=1, \text{allele from ancestry } A_1) + p(g=1, \text{allele from ancestry } A_2) \\ &= p(g=1|\text{allele from ancestry } A_1)p_a + p(g=1, \text{allele from ancestry } A_2)(1-p_a) \\ &= p_1 p_a + p_2(1-p_a) = (p_1 - p_2)p_a + p_2 \end{aligned}$$

Therefore, power for association analysis is based on allele frequency $(p_1 - p_2)p_a + p_2$ and effect size β, while power for admixture mapping is based on allele frequency p_a and effect size $(p_1 - p_2)\beta$. Note that if the same p-value threshold was used for determining significance in association and admixture mapping, then association mapping would have always been more powerful than admixture mapping (given that the genotypes was available in association mapping). However, power advantage for admixture mapping comes from lower p-value threshold. This power advantage is present when local allele frequency is both relatively high (close to 0.5), $|p_1 - p_2|$ is relatively high, and the variant effect is relatively high, as seen in Figure H.

5.1 Additional power considerations

More complex scenario, e.g. different genotype effects between ancestries, multiple local ancestries, and multiple causal variants in the LAI are plausible but are harder to derive analytically due the the increase in the number of free parameters.

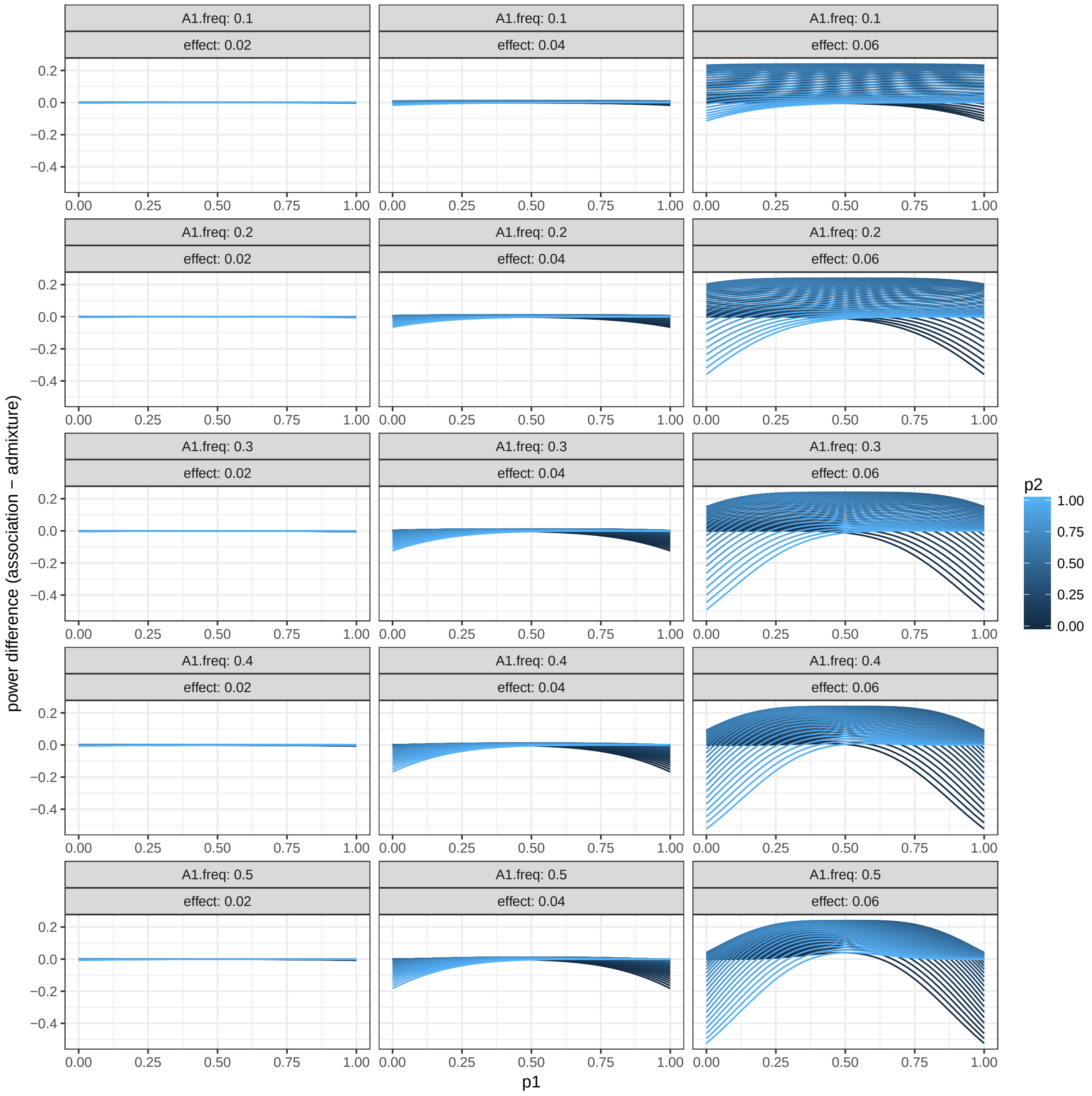

Figure H: The power difference between association and admixture mapping for given values of frequencies p_a of ancestry 1 (A_1), effect size in standard deviation of the outcome, MAF of the single causal variant in A_1 p_1, MAF of the causal variant in A_2 p_2, and based on $n = 12,500$ and p-value threshold for significance 5×10^{-8} in association mapping, and 5.68×10^{-5} in admixture mapping. Positive values indicated larger power for association mapping, and negative values indicate larger power for admixture mapping.
